# Self-help management of side effects in female cancer patients -analysis of the forum of the Women’s Self-help Association against cancer

**DOI:** 10.1186/s12885-025-14816-3

**Published:** 2025-09-09

**Authors:** Louisa Schäfer, Lena Josfeld, Jutta Hübner

**Affiliations:** Klinik für Innere Medizin II, Universitätsklinikum Jena, Am Klinikum 1, Jena, 07747 Germany

**Keywords:** Breast cancer, Gynaecological cancer, Self-help groups, Patient information, Digital forum

## Abstract

**Background:**

Side effects of cancer treatment have a high impact on patients. Besides physicians and nurses, other patients are an important partner for communication on side effects. Online fora within self-help groups offer a digital option for this communication. Our aim was to assess the content of the largest German self-help forum for cancer patients with respect to side effects of cancer treatment.

**Methods:**

All threads in the self-help forum of the German women’s self-help (11/17/2012 to 06/01/2021) under the topic “side effect management”- “how can I help myself?” were assessed quantitatively (topic, duration, number of answers at all and per day at peak) and regarding their content.

**Results:**

The vast majority of patients mentioned different kinds of pain as side effects, followed by infection or necrosis. While most threads showed a target-oriented search to obtain specific information, a quarter also dealt with strong emotions (anxiety, worry, despair). The main goal in starting a thread was to find information and other patients with similar problems. Most reactions (visits and answers) were received by threads on side effects of chemotherapy.

**Conclusions:**

The forum of a self-help group allows interactive patient-centred discourse between members independent of place and time. It provides insight into the meaning and burden of side effects from the patients’ perspective. This may help to optimise the support of cancer patients.

## Introduction

There is a strong tendency for patients and caregivers to search for help in specialised platforms on the Internet after getting a diagnosis of cancer [1].

Many different online self-help groups are available, and in particular, younger patients engage in peer-to-peer access through online communities, often even despite the lack of conclusive evidence for any positive effect on patient-reported outcomes. The main reason appears to be the hope to share not only information but also emotion and sometimes strategies for coping with and living with the diagnosis [2, 3].

Cancer treatment and its side effects have a high impact on the daily life of the patient bringing various limitations. Self-efficacy “as the ability for persons to maintain health-related behaviour change” seems to be a promising aspect to improve symptoms of cancer therapy. The participation in the forum helps to reduce social isolation and promotes higher self-efficacy [4].

We expected well-known chemotherapy side effects like nausea and emesis to play a central role in the patients’ communication. This type of self-help, which in former times was mostly based on local groups with weekly group meetings, has developed into powerful organisations of local and online support [1].

The Women’s Self-help Association against Cancer (Frauenselbsthilfe Krebs) is the most famous self-help group in Germany for women diagnosed with cancer. Further details about the organisation are provided in the Materials section.

In many online communities, quality of the content is heterogeneous and false information may rapidly spread [5].

Consequently the forum of the Women’s Self-help Association against Cancer has several moderators, who monitor compliance with the netiquette, concerning content (no advertising) and style (respectful conversations).

The objective of our study was a detailed analysis of the dialogue content in the side effect section; therefore we aimed at structuring the vast number of threads with regard to prevalence of side effects of cancer treatment, emotions and aim of the authors, which start a thread. The results of our study add to the research field a provisioning of information for physicians and nurses for a better communication with their patients.

## Materials and methods

### Material

With permission from the Women’s Self-help Association against Cancer (Frauenselbsthilfe Krebs), the oldest and largest self-help organization in Germany, we assessed the chapter on side effects [5]. The Frauenselbsthilfe Krebs was first founded in 1976 as a local group by 15 women after ablatio mammae and evolved into a national self-help network with about 30,000 members over the years [6]. Around 8,500 of the members of the association, which is supported by the German Cancer Aid organisation (Deutsche Krebshilfe), are connected via an Internet forum where people directly or indirectly affected by cancer can not only inform themselves, but also correspond with each other.

The Frauenselbsthilfe is open to female patients with all types of cancer and for men with breast cancer, with most members having breast or gynaecological cancer. In 2012, a digital forum was started. The access to all threads of this forum is free. Users have the possibility to create their own profile and can open threads or answer to threads of all topics. All data of the member profile is voluntary. At the end of the survey period (06/01/2021) the forum included about 415,000 starting posts and replies and had 8,500 members. Our analysis of 150 threads represents approximately 1.8% of the total threads available on the platform.

The forum is organized by volunteers who themselves are patients and experienced members of the organization. They work as moderators using a netiquette policy which aims at keeping off false information, advertising or aggressive or offensive speech.

On the forum’s main page, there is a classification of the topics into “General” (e.g. round of introductions, encouragement stories) and “Our topics” (Type of cancer, methods of treatment, how can I help myself?).

In the present work we analysed threads about side effects of cancer treatments which can be found in the category “Side effect management (e.g. fatigue)” following the forum’s main page under “Our topics” and “How can I help myself?”

We included threads that have been published from 11/17/2012 to 06/01/2021.

## Methods

Two scientists of our team accessed the digital forum and started with a quantitative analysis comprising title of the topic, number of answers, number of visits, start date, date of the last post before 06/01/2021, duration of the conversation of the thread in days, answers per day, visits per day.

In a second step our team created a classification by topic of the starting post, type of side effect, emotion, attitudes and aim. We defined the first mentioned symptom in the starting post as the side effect most prominently mentioned in the starting post (Fig. 2) and all following symptoms as side effects mentioned in the starting post in second ranking (Fig. 3).

The emotion section has been analysed by two authors (LJ and JH). They noted each emotion according to Plutchik`s wheel of emotions. In case of dissent a discussion took place and a consensus has been found.

We defined consistent terminology for our analysis: thread is a string of messages including starting post and answers. The starting post is the first message in a thread and the headline of the starting post is also the topic of the thread. The starting post is followed by answers from users of the forum and in some cases also by questions or remarks of the user who wrote the starting post. Our systematic content analysis focused specifically on topics mentioned in starting posts, while thread engagement metrics reflect overall thread activity including responses.

Statistical comparisons between groups were performed using Kruskal-Wallis tests due to non-normal distribution of metrics (Shapiro-Wilk test, *p* <.001). Post-hoc analyses used Dunn’s test with Bonferroni correction. Statistical significance was set at *p* <.05. All analyses were performed using appropriate statistical software.

## Results

In total, 150 threads between 11/17/2012 and 06/01/2021 were captured and systematically analysed. Of these three threads were started by the daughters, two for their mothers, who were patients with breast cancer. For the other (being a father) the type of cancer cannot be concluded from the text. All others were started by patients themselves. Most patients had breast cancer (*N* = 98; 65%) and 5 (3%) had gynaecological cancers. For 40 patients (27%) the type of cancer cannot be determined from the data in the text (direct diagnosis or information on the part of body involved or type of typical treatment). Three patients had lymphoma (2%), one a carcinoma of the urethra, one a nasopharynx carcinoma and one stomach cancer (1% each). One patient wrote that most probably she had a benign tumour.

### Topics mentioned in the starting posts

**Fig. 1:**
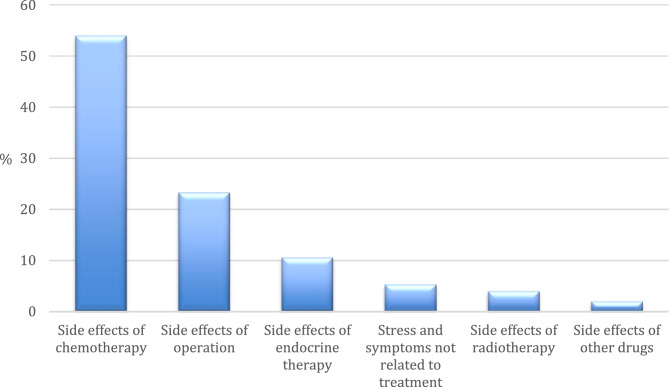
Topics mentioned in starting posts (*N* = 150)

Concerning the topic mentioned in starting posts (Fig. 1), most dealt with side effects of treatment (*n* = 137, 91%), mostly due to chemotherapy (*n* = 81, 54%). Side effects of other drugs were mentioned in three starting posts (twice cortisone, one opioids).

### Side effects mentioned in the starting posts

**Fig. 2:**
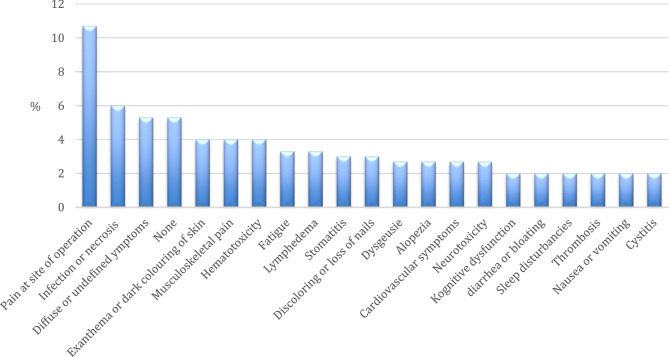
Side effects prominently mentioned in starting posts (*N* = 150)

**Fig. 3:**
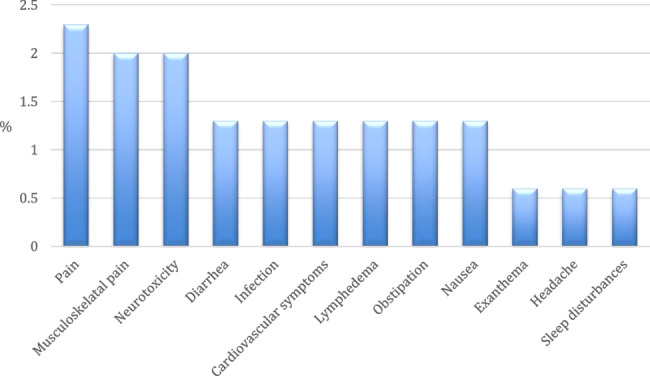
Side effects mentioned in starting posts in second ranking (*N* = 150)

We distinguished between the prominent side effect or symptom in the starting post (Fig. 2) and other symptoms frequently mentioned in second ranking (Fig. 3).

The type of side effects varied widely and 37 threads (25%) dealt with side effects mentioned only in one or two starting posts. Pain emerged as the most frequently mentioned side effect in the starting posts. Specifically, pain at the point of surgery was the most prominent side effect (*n* = 16, 11%), followed by musculoskeletal pain (*n* = 6, 4%). These types of pain were also the most commonly mentioned secondary symptoms, with post-operative pain and musculoskeletal pain mentioned in 5 (3%) and 3 (2%) posts respectively.

### Emotions and attitudes mentioned in starting posts

**Fig. 4:**
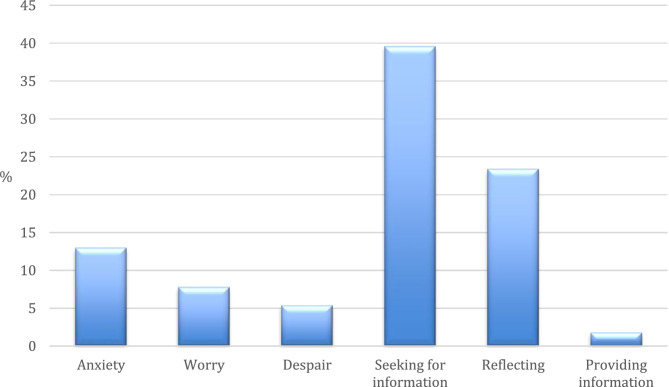
Emotions and attitudes mentioned in starting post (*N* = 150)

Some starting posts clearly named an emotional reaction, which was described in the text (Fig. 4). Strong emotions were shown in 39 posts (26%). The most frequent emotion was anxiety (*n* = 19, 13%), followed by worries (*n* = 12, 8%) and despair (*n* = 8, 5%). The majority of the starting posts are target-oriented and we could not detect any emotion according to Plutchik’s wheel of emotions. Some of the aforementioned starting posts were describing side effects and reflecting on different strategies to reduce these problems (*n* = 35, 23%). Most starting posts were asking for information and hints (*n* = 59, 40%) and only very few were written to inform other patients of solutions found (*n* = 3, 2%).

### Aims of starting posts

**Fig. 5:**
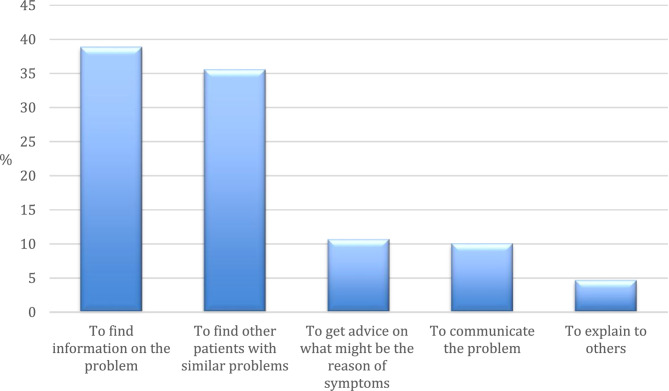
Aims of starting posts (*N* = 149; one starting post only asked another patient on how she felt)

The starting posts aimed at different goals (Fig. 5). Most often, the patient was looking for information asking other patients on where to find the information or on their personal tips/ideas (*n* = 58, 39%). Almost as frequently the patients were looking for other patients who had experienced the same problems (*n* = 53, 36%). Getting advice on what might be the cause of symptoms as well as writing down and communicating the problem was the intention in *n* = 16, 11% and *n* = 15, 10% of the starting posts respectively.

### Comparison of the threads

We analysed the metric data of 150 forum threads focusing on side effects, emotions, and aims. Overall thread metrics showed substantial variation in engagement: duration (M = 959 days, SD = 487, range: 371–1906), responses (M = 61, SD = 43, range: 18–232), and visits (M = 19,100, SD = 12,500, range: 6,750 − 58,650). Among treatment-related topics, chemotherapy threads showed highest engagement (duration: M = 1,200 days, SD = 523; responses: M = 87, SD = 46; visits: M = 26,200, SD = 13,900), significantly exceeding other treatment categories (*p* <.05, Kruskal-Wallis test). Radiotherapy threads demonstrated notably lower metrics (duration: M = 518 days, SD = 313; responses: M = 22, SD = 19; visits: M = 6,750, SD = 5000).

Regarding specific side effects, neurotoxicity threads showed highest engagement metrics (duration: M = 1,900 days, SD = 679; responses: M = 232, SD = 112; visits: M = 58,700, SD = 23,500), while diffuse symptoms had shortest duration (M = 371 days, SD = 198) but highest response rate (1.6 responses/day, SD = 0.9).

Emotional content analysis showed anxiety-related threads generated significant engagement (responses: M = 87, SD = 43; visits: M = 28,300, SD = 15,700). Posts seeking symptom causes showed highest daily engagement (responses/day: M = 1.0, SD = 0.6; visits/day: M = 24.9, SD = 12.5) (Tables 1, 2, 3 and 4).

**Table 1:** Overall thread metrics (*N* = 150)

Metric	Mean ± SD	Median	Range
Duration (days)	959 ± 487	903	371–1906
Number of answers	61 ± 43	50	18–232
Answers per day	0.8 ± 0.4	0.6	0.1–1.6
Number of visits	19,100 ± 12,500	13,500	6,750 − 58,650
Visits per day	12.7 ± 8.5	11.2	5.7–24.9

**Table 2:** Treatment-related side effects metrics

Treatment Type	Duration (days)	Answers	Visits	Answers/day	Visits/day
Chemotherapy	1,200 ± 523^*^	87 ± 46^*^	26,200 ± 13,900^*^	0.5 ± 0.2	15.4 ± 9.1^*^
Endocrine treatment	653 ± 335	23 ± 19^†^	10,500 ± 7,700	0.4 ± 0.2	13.0 ± 7.7
Operation	787 ± 412	39 ± 26	12,800 ± 8,800	1.2 ± 0.7^*^	9.3 ± 5.4
Radiotherapy	518 ± 313^†^	22 ± 19^†^	6,750 ± 5000^†^	0.3 ± 0.2^†^	5.7 ± 3.5^†^

^*^Significantly higher than other categories (*p* < .05)

^†^Significantly lower than other categories (*p* < .05)

**Table 3:** Specific side effects metrics

Side Effect Type	Duration (days)	Answers	Visits	Answers/day	Visits/day
Pain after operation	772 ± 390	39 ± 25	13,200 ± 9000	1.1 ± 0.5^*^	11.0 ± 6.8
Musculoskeletal pain	1,460 ± 568^*^	127 ± 79^*^	46,200 ± 21,300^*^	0.8 ± 0.5	21.4 ± 11.2^*^
Neurotoxicity	1,906 ± 679^*^	232 ± 112^*^	58,650 ± 23,500^*^	0.1 ± 0.1^†^	24.0 ± 12.3^*^
Diffuse symptoms	371 ± 198^†^	18 ± 12^†^	6,850 ± 4,600^†^	1.6 ± 0.9^*^	5.9 ± 3.5^†^
Lymphedema	949 ± 457	59 ± 35	12,800 ± 7,700	0.2 ± 0.1^†^	7.5 ± 4.6

^*^Significantly higher than other categories (*p* < .05)

^†^Significantly lower than other categories (*p* < .05)

**Table 4:** Emotions and thread purpose metrics

Category	Duration (days)	Answers	Visits	Answers/day	Visits/day
Emotions/attitudes					
Anxiety	874 ± 423	87 ± 43^*^	28,300 ± 15,700^*^	0.2 ± 0.2^†^	16.3 ± 9.2^*^
Worry	1,089 ± 499	45 ± 29	22,500 ± 13,500^*^	0.5 ± 0.3	18.9 ± 10.2^*^
Despair	912 ± 446	85 ± 46^*^	18,900 ± 11,200	0.3 ± 0.2^†^	11.4 ± 6.8
Thread Purpose					
Finding information	1,138 ± 512	60 ± 32	19,100 ± 11,200	0.9 ± 0.5	10.1 ± 5.7
Seeking similar cases	852 ± 412	59 ± 35	19,800 ± 12,300	0.6 ± 0.3	12.0 ± 6.9
Getting symptom advice	855 ± 423	106 ± 57^*^	29,000 ± 15,700^*^	1.0 ± 0.6^*^	24.9 ± 12.5^*^
Communicating problem	833 ± 399	32 ± 21^†^	10,600 ± 6,800^†^	0.2 ± 0.2^†^	13.4 ± 7.9

^*^Significantly higher than other categories (*p* < .05)

^†^Significantly lower than other categories (*p* < .05)

## Discussion

Our analysis shows that self-help group forums serve as vital platforms for patient information exchange and emotional support. Several key findings emerge from our investigation.

### Diversity of side effect discussion

The forum demonstrates remarkable diversity in side effect discussion, with most threads addressing currently experienced symptoms rather than anticipated ones. While van Eenbergen et al. [3] reported general diversity in online cancer communities addressing patient-reported outcomes beyond clinical measures, our analysis specifically quantified this diversity with 37 different side effects mentioned in starting posts, with 25% of threads dealing with side effects mentioned only once or twice. This demonstrates even greater diversity than previously documented in the literature.

Notably, the most frequently mentioned side effects differed from those typically emphasized in clinical guidelines and physician training.

While chemotherapy-related discussion dominated, the focus was not on commonly cited side effects like nausea and emesis, but rather on impacts affecting appearance of a person (skin, hair, nails) and bodily functions (sense of touch, taste). This contrast between our forum findings and clinical emphasis confirms the discrepancies between physician and patient priorities reported in previous research [7, 8], suggesting that physician-patient communication about side effects should extend beyond standard guideline-focused discussions to address individual patient concerns.

### Pain management gaps

Our analysis showed pain as the predominant concern across treatment types, with post-surgical pain (11% of starting posts) and musculoskeletal pain (4%) being the most frequently mentioned issues in starting posts.

This high prevalence in our forum data correlates with clinical studies showing pain as an underaddressed concern in cancer care [7]. The gap between our findings and expected clinical management points to a potential deficit in supportive treatment, particularly noteworthy since pain is a side effect for which many treatment options exist, including analgesics, physiotherapy, and massage therapy.

The reasons for this deficit appear heterogeneous based on the forum discussions we analyzed. Several posts indicated a lack of communication between patients and physicians, though it remains unclear whether this stems from patients not reporting pain or physicians not adequately addressing reported symptoms. Our forum analysis corroborates previous studies by Wuensch et al. [7] and Jung et al. [8], with patients reporting insufficient discussion of side effects and limited information about supportive treatment options. This communication gap is particularly concerning as patients who are not informed about treatment options may not contact their physician when experiencing side effects.

These findings from our starting post analysis suggest two key areas for improvement: enhanced patient education about pain management options and implementation of routine screening for side effects. A standardized screening instrument, including options for free-text responses, could help bridge the current communication gap between patients and healthcare providers, as suggested by previous research on patient-provider communication in cancer care [7, 8] and supported by the information-seeking behavior we observed in 39% of starting posts.

### Musculoskeletal effects

The high engagement metrics for musculoskeletal side effects - including sustained thread duration (M = 1462 days), numerous responses (M = 127), and frequent daily visits (M = 46,200) - suggest these symptoms represent an ongoing challenge for patients. Our quantitative thread analysis data correlates with clinical observations about the persistent nature of musculoskeletal complaints in cancer patients.

This persistent discussion activity in starting posts and subsequent thread responses particularly highlights the importance of addressing endocrine therapy-related side effects, as they can lead to treatment non-adherence and consequently reduced survival outcomes, as documented by Wuensch et al. [7] in their study of breast cancer patients.

### Neurotoxicity concerns

Threads about neurotoxicity demonstrated the highest engagement metrics across all measures, indicating this represents a particularly challenging side effect for patients with limited treatment options. In fact, supportive treatments for this common side effect of taxane-based treatments rarely exist and recommendations in guidelines are scarce [9]. This again points to an significant lack of physician/patient communication which needs to be urgently addressed.

### Forum usage patterns

Our analysis of starting posts showed two primary user intentions. Information-seeking or reflection on side effects accounted for nearly two-thirds of the threads (combining 39% seeking information and 23% reflecting on strategies). Less frequent were threads prominently describing emotional reactions to side effects and searching for emotional support from other users (26% showing strong emotions in starting posts).

Huber et al. [10] demonstrated that online support groups have a significant role in patients’ treatment decision-making and social environment for prostate cancer patients, with information-seeking as a key motivation. Our findings align with this, showing 39% information-seeking posts, but our data additionally shows 36% seeking similar cases, indicating a more nuanced usage pattern than previously reported. While Huber et al. focused on treatment decision-making impacts, our analysis demonstrates that cancer patients use forums for both information gathering and peer identification simultaneously.

Similarly, Bender et al. [11] observed that online community users primarily sought information and symptom management support, with emotional support as a secondary. Our quantitative data confirms this hierarchy (39% information-seeking vs. 26% emotional content in starting posts), but shows a smaller gap between information and emotional needs than Bender et al. reported. This suggests that patients turn to online communities “to address unmet needs during periods of stress and uncertainty” [11], with our forum showing more balanced integration of practical and emotional support compared to earlier studies.

While Sandaunet [12] highlighted online self-help groups as promising e-health tools for coping with health problems, our analysis provides concrete quantitative evidence supporting this claim through the sustained engagement we observed (mean duration 959 days), particularly around complex treatment side effects mentioned in starting posts. However, while Sandaunet focused on participation challenges, our forum showed remarkably sustained engagement, suggesting that well-moderated cancer forums may overcome the participation barriers she identified.

### Future implications

Traditional cancer therapies are now complemented by new treatments such as oral anticancer agents (OAAs), which often require extended treatment periods beyond initial therapy. These newer treatments present specific challenges, as patients and caregivers must manage complex issues including side effects and drug interactions. The increasing complexity of these treatment regimens suggests a growing need for support from both healthcare professionals and self-help fora. Self-help forums represent a valuable resource for future research into new cancer treatments, offering insights into patient expectations and needs. Our study contributes to the existing body of research by analyzing authentic patient discourse in side effect management without researcher intervention that might influence responses, such as interviews or questionnaires.

### Limitation

Our research has several important limitations. The scope is limited as it only analysed the side effect category of the forum. This restriction to medical topics may introduce bias, as we cannot assess how side effect discussions might differ when integrated with broader life concerns mentioned in other forum categories. Additionally, expanding the analysis to include other categories would not lead to more detailed information about side effects but to broader information about overall patient experiences, which was beyond the scope of this study.

Further population restrictions are demonstrated. The forum of the Women’s Self-help Association against Cancer only includes gynaecological cancer, most of the users are breast cancer patients and female. Other subgroups in the population of cancer sufferers were not addressed.

Additionally, the study’s methodological limitations encompass the subjective assessment of emotions by two researchers, self-selection bias in forum participation, and lack of verified demographic data from participants. Our analysis of 150 threads represents approximately 2% of the total threads available on the platform, which may limit the generalizability of our findings to the broader forum community.

## Conclusions

Our analysis shows that patients have a high need to communicate about pain and other side effects of cancer treatment. While patients are accompanied by healthcare professionals during therapy, they significantly benefit from peer exchange. The self-help forum provides various benefits including maintaining social integration, recognizing personal resources, overcoming fears, improving quality of life, and developing informed self-advocacy.

Our findings suggest that physicians should incorporate moderated self-help forums into their patient care recommendations, as these platforms facilitate patient-physician communication and provide valuable patient-related outcomes. However, these forums should serve as a complement to, not a substitute for, other patient information materials. Understanding these patient perspectives is crucial for improving cancer treatment and should be a priority for oncology practitioners.

## Data Availability

All data generated or analysed during this study are included in this published article.
